# Supplementary material for: Are Signals Regulating Energy Homeostasis Related to Neuropsychological and Clinical Features of Gambling Disorder? A Case–Control Study
Source: Nutrients. 2022 Nov 29;14(23):5084. doi: 10.3390/nu14235084 (PMC9736671; doi:10.3390/nu14235084)
Supplement: Supplementary file 1 [file nutrients-14-05084-s001.zip › nutrients-2010011-supplementary.pdf]

Table S1. Characteristics of the sample.

		Control (*N* = 41)		GD (*N* = 297)		
		n	%	*n*	%	*p*
Sex	Women	4	9.8%	19	6.4%	0.423
	Men	37	90.2%	278	93.6%	
Education	Primary	7	17.1%	157	52.9%	**<0.001 ***
	Secondary	15	36.6%	112	37.7%	
	University	19	46.3%	28	9.4%	
Marital	Single	17	41.5%	158	53.2%	**0.014 ***
	Married	23	56.1%	103	34.7%	
	Divorced	1	2.4%	36	12.1%	
Social index	High	0	0.0%	8	2.7%	**0.005 ***
	Mean-high	2	4.9%	19	6.4%	
	Mean	3	7.3%	24	8.1%	
	Mean-low	28	68.3%	113	38.0%	
	Low	8	19.5%	133	44.8%	
		Mean	SD	Mean	SD	*p*
Age (years-old)		49.27	15.23	39.58	14.16	**<0.001 ***
BMI (kg/m²)		24.79	1.98	26.48	5.04	**0.035 ***

		GD (*N* = 297)	
		Mean	SD
Age of onset of GD		29.10	12.42
Duration of GD		5.23	6.02
Number DSM-5 criteria for GD		7.13	1.80
		n	%
Debts due to GD	No	122	41.1%
	Yes	175	58.9%
Gambling	Non-stratetigic	148	49.8%
	Strategic	92	31.0%
	Mixed	57	19.2%
Substances use		*n*	%
Tobacco	Yes	138	46.5%
Alcohol	Yes	43	14.5%
Other drugs	Yes	34	11.4%

Note. GD: gambling disorder. BMI: body mass index. SD: standard deviation. DSM-5: Diagnostic and Statistical Manual of Mental Disorders, Fifth Edition. * Bold: significant comparison.

Table S2. Partial correlation matrix.

	Control (*N* = 41)				Gambling disorder (*N* = 297)			
	**Ghrelin**	**LEAP2**	**Leptin**	**Adeno.**	**Ghrelin**	**LEAP2**	**Leptin**	**Adeno.**
[1] Psychopathology: SCL-90R								
Somatization	−0.088	0.220	0.128	−0.101	0.064	0.092	0.042	0.018
Obsessive/compulsive	0.119	0.047	−0.143	−0.079	0.043	0.141	0.084	0.046
Interpersonal sensitivity	0.173	**0.266** †	0.043	−0.009	0.050	0.026	−0.028	0.028
Depressive	0.135	0.134	−0.116	0.048	0.073	0.056	0.040	−0.037
Anxiety	0.061	**0.271** †	0.032	0.073	0.018	0.122	0.046	−0.019
Hostility	−0.065	0.058	0.020	0.064	0.070	0.055	−0.013	−0.006
Phobic anxiety	0.126	**0.317** †	**0.237** †	−0.119	0.023	0.057	0.119	0.082
Paranoid Ideation	0.199	**0.304** †	0.097	−0.030	0.011	0.104	−0.021	0.021
Psychotic	0.168	0.192	0.063	0.079	0.028	0.074	0.023	0.004
GSI score	0.093	0.226	−0.016	0.001	0.054	0.092	0.033	0.010
PST score	0.151	0.179	−0.031	−0.069	0.042	0.063	0.018	0.004
PSDI score	−0.075	**0.370** †	0.093	0.155	0.057	0.080	0.016	−0.013
[1] Impulsivity: UPPS-P								
Lack premeditation	0.012	−0.064	0.089	**−0.290** †	0.040	−0.018	−0.028	0.024
Lack perseverance	0.029	−0.184	−0.098	−0.150	0.009	0.013	0.033	0.066
Sensation seeking	−0.079	−0.234	−0.123	−0.004	−0.013	−0.009	−0.101	0.058
Positive urgency	−0.055	−0.120	0.006	−0.183	0.028	0.096	−0.074	0.004
Negative urgency	−0.091	0.132	0.126	−0.159	0.050	0.073	−0.055	0.019
Total	−0.068	−0.146	−0.010	−0.207	0.027	0.053	−0.076	0.050
[1] Gambling severity								
DSM-5 criteria	−0.073	0.141	0.073	−0.126	0.029	0.013	0.081	−0.041
[2] Neuropsychological data								
IGT: block 1	0.150	0.070	0.003	−0.002	0.009	0.091	−0.037	0.054
IGT: block 2	0.005	0.141	−0.058	0.095	−0.013	−0.002	−0.052	0.034
IGT: block 3	0.020	0.008	**−0.263** †	−0.075	0.020	0.062	0.011	0.047
IGT: block 4	0.005	0.075	**−0.345** †	−0.005	−0.025	0.043	−0.081	0.017
IGT: block 5	**−0.258** †	−0.096	−0.224	−0.071	−0.181	0.039	0.029	−0.107
IGT: total	−0.044	0.034	**−0.280** †	−0.035	−0.074	0.071	−0.033	0.001
IGT: learning	−0.191	−0.077	**−0.331** †	−0.076	−0.124	0.014	0.009	−0.090
IGT: risk	−0.130	−0.003	**−0.330** †	−0.040	−0.127	0.048	−0.026	−0.057
WCST: trials	**−0.317** †	0.127	−0.006	0.008	0.005	−0.063	0.042	−0.054
WCST: errors	−0.215	0.164	0.100	0.025	0.002	−0.065	−0.023	−0.090
WCST: errors perseverant	**−0.253** †	0.189	0.089	0.028	0.025	−0.066	−0.011	−0.100
WCST: conceptual	−0.154	−0.082	**−0.272** †	−0.109	−0.002	0.036	0.077	0.060
WCST: categories completed	0.164	−0.053	−0.126	0.002	−0.038	0.019	0.054	0.040
WCST: trials complete 1-cat.	0.005	0.065	−0.124	0.061	0.046	−0.014	−0.023	−0.091
TMT: A	−0.103	**0.385** †	−0.062	0.061	−0.008	0.047	−0.030	0.025
TMT: B	−0.133	**0.368** †	**0.295** †	0.034	−0.066	0.036	−0.036	0.108
TMT: Diff	−0.118	**0.282** †	**0.389** †	0.015	−0.066	0.029	−0.032	0.110
Stroop: words	0.082	−0.195	0.110	0.103	−0.066	−0.027	0.053	−0.075
Stroop: colors	−0.050	−0.160	−0.035	−0.099	−0.031	−0.085	0.004	−0.085
Stroop: words-colors	−0.052	**−0.340** †	0.067	0.024	−0.027	−0.032	−0.023	−0.008
Stroop: estimated	−0.008	−0.192	0.033	−0.032	−0.050	−0.068	0.027	−0.091
Stroop: interference	−0.065	**−0.316** †	0.066	0.057	0.000	0.006	−0.050	0.055
WMS-III: direct	0.041	**−0.271** †	−0.232	**−0.289** †	−0.026	−0.134	0.021	−0.040
WMS-III: direct-span	0.026	−0.151	−0.103	−0.190	−0.036	−0.128	0.009	−0.067
WMS-III: inverse	0.028	−0.061	−0.111	−0.207	−0.075	−0.064	0.041	0.002
WMS-III: inverse-span	0.052	−0.172	−0.060	−0.195	−0.063	−0.070	0.049	0.031
WMS-III: total	0.040	−0.195	−0.200	**−0.289** †	−0.056	−0.110	0.035	−0.021
WAIS: vocabulary	−0.237	−0.003	0.008	0.046	0.050	−0.014	0.012	0.054

Note. [1] Partial correlation adjusted by sex, age, and BMI. [2] Partial correlation adjusted by sex, age, BMI and education level. LEAP2: liver enriched antimicrobial peptide 2. SCL-90R: Symptom Checklist-90-Revised. GSI: Global Severity Index. PST: Positive Symptom Total. PSDI: Positive Symptom Distress Index. UPPS-P: Impulsive Behavior Scale. IGT: Iowa Gambling Test. WCST: Wisconsin Card Sorting Test. TMT: Trail Making Test. WMS-III: Wechsler Memory Scale Third Edition. WAIS: Wechsler Adult Intelligence Scale. † Bold: effect size into the range mild-moderate (|*R*| > 0.24) to high-large (|*R*| > 0.37).
